# Fumonisin Distribution in Maize Dry-Milling Products and By-Products: Impact of Two Industrial Degermination Systems

**DOI:** 10.3390/toxins10090357

**Published:** 2018-09-04

**Authors:** Francesca Vanara, Valentina Scarpino, Massimo Blandino

**Affiliations:** Dipartimento di Scienze Agrarie, Forestali e Alimentari, Università degli Studi di Torino, Largo Paolo Braccini, 2, 10095 Grugliasco, Italy; francesca.vanara@unito.it (F.V.); valentina.scarpino@unito.it (V.S.)

**Keywords:** reduction, mycotoxins, tempering-degermination, dry-degermination, milling fractions

## Abstract

In temperate areas, the main limitation to the use of maize in the food chain is its contamination by B-series fumonisins (FBs) during cultivation. Since the content of this group of mycotoxins may be distributed unevenly after milling, the aim of this study was to compare the distribution of FBs in maize fractions derived from two industrial dry-milling processes, that is, a dry-degermination (DD) system and a tempering-degermination (TD) system. Grain cleaning reduces FBs by about 42%. The germ of the two degermination processes showed a similar FB content of kernel after cleaning. Conversely, an animal feed flour resulted in a FB content that was two times higher than whole grain before cleaning. A significant FB reduction was observed in the milling fractions in both processes, with a higher reduction in the TD system than in the DD one. The average decontamination respect to uncleaned kernels in the DD process was 50%, 83% and 87%, for maize flour, break meal and pearl meal, respectively, while it was 78%, 88% and 94% in the TD process for small, medium and flaking grits, respectively. Among the milling fractions, the flaking grits with the highest particle size resulted in the highest FB reduction.

## 1. Introduction

Milling processes are methods that can be used to transform whole grains into forms suitable for conversion into consumable products. They usually separate the botanical tissue of the grain and reduce the endosperm into flour or grits [1].

From the processing perspective, the maize kernel is composed of four primary structures: the endosperm, germ, pericarp and tip cap, and they generally make up 83%, 11%, 5% and 1% of the maize kernel, respectively [2]. The endosperm is mainly made up of starch surrounded by a protein matrix. There are two types of endosperm and these influence grain hardness: floury (soft or mealy) or horny (hard or vitreous), which depend on the size, morphology and compaction grade of the starch granules and the nature of the protein matrix. The type of proteins in particular are the determining factor that can explain endosperm vitreousness [3].

Most of the maize used for food is first processed by either wet or dry milling industries. Wet milling fundamentally differs from dry milling in that it is a maceration process in which physical and chemical changes occur in the nature of the basic constituents. Wet milling produces pure starch for industrial and food uses, and by-products composed of protein, fibre and germ [1].

Dry-milling is the main milling procedure adopted in the maize food chain, and it produces refined endosperm products with various particle sizes and other by-products such as germ and animal feed flour [4]. This process could be carried out with tempering-degermination (TD) or without dry-degermination (DD) a tempering step before degermination. Tempering is done adding water in order to create differential swelling resulting from the higher absorbing moisture of germ and pericarp, that lead to a loss of tissue connection between the germ and the endosperm and a faster removal of these fractions [5]. The heart of the TD process is a Beall-type degerminator that produce a high yield of large particle size grits (flaking grits, hominy grits) with low-fat content, suitable for manufacture of corn flakes and other extruded transformations [4], although further grinding and refining processes could be applied to these products in order to obtain maize meal and flour. Otherwise, in the DD system, maize grains are broken by an impact degerminator, and this process is employed only for the production of maize meal and flour. High kernel hardness [6], with high incidence of horny endosperm and low incidence of stress cracks [4], related mainly to high temperature during the drying process, are the major quality criteria for maize used in dry milling, in particular for TD.

Considering the increasing role of maize milled products as basic food, their quality characterization becomes extremely important. From a nutritional point of view, maize and its derived products are good sources of starch, proteins, lipids and different bioactive compounds [7,8]. However, the quality of maize could be reduced by the presence of mycotoxins and in particular by B-series fumonisins (FBs), a class of secondary fungal metabolites produced by *Fusarium* species from the *Liseola* section that are the most important mycotoxins found in maize grain in temperate areas [9,10]. As FB producer fungi grow over a wide range of temperatures, but only when there is a high water activity (a_w_ > 0.9), these mycotoxins are formed in maize prior to the harvest. B-series fumonisins are fairly heat-stable and their content is only significantly reduced during processes in which the temperature exceeds 150 °C [9]. Even when there is no detoxification, a re-distribution of FBs has been observed in milled products, with a very different content of these metabolites in the kernel components [11]. The mycotoxins may be distributed during the milling process, thus yielding higher or lower levels in the various milled products [12]. The control of the FBs content in the chain from the unprocessed grains to the processed maize-based foods is necessary considering also the European Commission Regulation (EC) No. 1126/2007 [13].

The re-distribution of FBs in maize dry milling has already been analysed extensively in other studies [14,15,16,17], but always considering separately a DD system or a TD degermination system. The aim of this study has been to investigate and compare the distribution of fumonisin B_1_ (FB_1_), fumonisin B_2_ (FB_2_) and FBs as a sum of FB_1_ and FB_2_ in milling products and by-products obtained from two degermination systems and from the processing of the same maize lots at the industrial commercial scale, in order to obtain a clear comparison of the decontamination levels obtainable by applying different dry-milling processes.

## 2. Results and Discussion

As far as the considered growing seasons are concerned, the average recorded FB contamination in pre-cleaned whole grain was 357 µg·kg^−1^, 1292 µg·kg^−1^, 2123 µg·kg^−1^, in 2011, 2012 and 2013 growing seasons, respectively (Table 1).

Analysis of variance (ANOVA) showed significant differences (*p* < 0.001) for both FB_1_ and FB_2_ and for their sum, between the milling fractions obtained considering both the compared dry-milling processes. Otherwise the interaction between the milling fraction and the year of production of the processed lots was never significant for FB contamination.

The effect of the cleaning operation on the FB concentration was significant, with a mean content in the pre-cleaned and post-cleaned whole grain of 1257 µg·kg^−1^ and 725 µg·kg^−1^, respectively, which corresponds to a content reduction of 42%. The abatement recorded in this production situation was on average higher than that observed for FB in previous studies with different cleaning steps (−10; −32% [18]; −32% [19]).

It is important to note that each commercial mill is likely to have its own particular set up, thus giving rise to different percentages of reduction, but a cleaning process is essential to obtain a healthy whole grain for milling.

The evaluation of the distribution of FBs in the maize kernel can start from the two botanical tissues that are usually separated at the beginning of the milling process: the germ and the bran. Even though two different degermination processes were considered, the effect on the FB contamination of the germ was the same. The mean FB content was 528 µg·kg^−1^ and 510 µg·kg^−1^ in the DD system and in the TD system, respectively. Germ contamination was not significantly different from that of the whole grain after cleaning (Table 2).

The animal feed flour, a mixture of bran and part of the mealy endosperm, was the by-product with the highest FB content (2.2 times higher in the DD process and 2.4 times in the TD process than the whole grain before cleaning). No significant differences were observed for the animal feed flour contamination of the compared processes.

Several previous scientific studies collected and analysed germ and animal feed flour together, and the results showed a 2 to 3 times higher FB content than the whole grain [15,20,21]. In other scientific studies [16,17], in the same way as in the present study, the germ was collected separately from the bran or the fine particles destined for animal consumption and it was always contaminated less than the pre-cleaned whole grain (−30% and −40%, respectively), while the animal feed flour was the highest contaminated fraction.

As a consequence of this unequal distribution of mycotoxins in the milling fractions, the products destined for human consumption were always significantly (*p* < 0.001) less contaminated than the germ and animal feed flour, and showed a variable re-distribution from the whole grain before cleaning in the different compared processes. The pearl meal, break meal and maize flour from the DD process had mean FB contents of 157, 216 and 623 µg·kg^−1^, respectively, which resulted in a decontamination, compared to the whole kernel before cleaning, of 87%, 83% and 50%, respectively. As far as the particle size effect is concerned, the FB content in the pearl and break meal was significantly lower (*p* < 0.001) than in the maize flour.

Considering the TD system, the mean FB content of the flaking grits and of the medium and small hominy grits was 76, 147 and 272 µg·kg^−1^, respectively. The FB reduction, compared to the whole grain before cleaning, was 94%, 88% and 78%, respectively. These fractions with different particle sizes significantly differed from each other as far as the FB content was concerned: compared to the flaking grits, the contamination was 1.9 and 3.6 times higher for the medium and small hominy grits, respectively.

From a comparison of the applied degermination processes, it is possible to state that the TD system achieved the best FB decontamination results for the flaking grits, which were significantly less contaminated: considering the percentage of reduction with respect to the whole grain before cleaning, the flaking grits showed 6% of the original kernel FB content, while the pearl meal and break meal instead showed a FB contamination that ranged from 12% to 17% with respect to the whole grain. When the single compounds, that is FB_1_ and FB_2_, were considered, the same percentage of reduction as that of the FBs was observed.

The milling yields and FB mass balance of each fraction are reported in Table 3 in order to facilitate the comparison of the considered systems. The mass balance, calculated for each fraction from the combination of the yield data during the milling process and the FB content, is a parameter able to describe how and in what percentage the mycotoxin is distributed in the fraction derived from maize kernel milling.

Considering the sum of the three products for human consumption obtained through TD process (flaking grits and medium and small hominy grits) the mean milling yield was 55%, while they only contributed to 6% of the total FB contamination of the whole grain. The pearl meal, break meal and maize flour from the DD dry milling system had a mean yield of 55%, and their FB content amounted to 9% of the total contamination of the kernel. These three products mainly originate from the horny endosperm, but a higher percentage of floury endosperm that is not completely separated by this process still remains especially in the maize flour [11].

Previous studies have overlooked the fumonisin redistribution with different milling process, whereas the present study has been specifically designed to directly compare the decontamination of FBs obtained through the application of different degermination and milling processes to the same lots of maize grain.

To compare the results of several scientific studies concerning the effect of maize milling on the distribution of mycotoxins, Table 4 summarizes the results of different degermination processes, under both experimental and industrial dry-milling conditions. The data are expressed as the percentage of FB remaining in each of the degermination milling fractions compared to the contamination of the whole kernel. All the milling fractions destined for human consumption are reported according to the description adopted by the authors, and when available, the particle size of each fraction is reported. All the studies pertaining to both of the considered degermination systems confirm the negative relationship between the particle size and the fumonisin content. The FB contamination of flaking grits derived from the TD processes ranges between 6 and 15%, compared to that of whole kernel. In the considered DD processes, the lowest occurrence of FBs was detected in the pearl and break meal and varied between 11 and 22%, compared to that of whole kernel. A reduction in the FB content of the maize flour was also observed, with respect to the whole grain, but the decontamination percentage fell between 40–60%.

Castelles et al. [21] attributed the high contamination rates in maize flour to the fact that this fraction is obtained from the fine particles that are mainly created during the milling of the external kernel layers. Other authors [16,20] instead considered the hypothesis that these high rates may be due to contamination of the flour with germ particles. Kent and Evers [1] and Gwirtz and Garcia-Casal [2] reported a negative relationship between the particle size of maize dry-milled fractions and their fat content. Thus, according to these authors, the main contribution of FB contamination to maize flour could originate from the floury endosperm located around the germ, where a higher development of fungal species is possible. Katta et al. [16] suggested that the fungus could primarily be located in the pedicel and germ, but Shim et al. [22] showed, through experimental data, that *F. verticillioides* produces only a few FBs in the germ. Therefore, even though the germ is not the best substrate for FB production, the floury endosperm around the germ could instead be a good place for the fungus that grows in the germ to produce mycotoxins. Philippeau et al. [23] reported that the starch in a vitreous endosperm is more encapsulated by prolamin (zein) proteins than in a floury endosperm, thus vitreous particles of maize kernels may be more resistant to microbial attack than floury ones.

Moreover, considering the amylose/amylopectin ratio of the maize endosperm, Dombrink-Kurtzman and Knutson [24] concluded that there is a higher amylopectin content in the soft endosperm than in the hard endosperm. Bluhm and Woloshuk [25] observed that the occurrence of amylopectin during kernel development induces FB_1_ biosynthesis, possibly through the uptake of α-1,6 linked glucosides, such as dextrin. Field experiments conducted in Italy [26] showed that waxy maize hybrids, which are basically constituted by amylopectin, were more contaminated with fumonisin than conventional hybrids.

Overall, the collected data underline how the floury endosperm was more contaminated than the horny one, and that the different effectiveness of degermination systems to separate these fractions could lead to a different decontamination capacity in the derived products. Thus, the TD system process, which is able to better separate the horny endosperm from finer fractions [1], permits a higher decontamination level to be obtained than the DD system. It is important to underline that several industrial mills, including the one considered in this study, apply a TD system with a Beall degerminator to produce grits that are subsequently refined into pearl and break meal and maize flour. In this case, the use of TD systems is not aimed at obtaining flaking grits, but at increasing the decontamination level in the derived meal and flour fractions.

Considering European legislation regarding fumonisins (sum of FB_1_ and FB_2_) [13], different maximum levels are set for smaller and larger milling fractions than 500 µm; these levels reflect the contamination level of the different fractions and are 2000 and 1400 µg kg^−1^, respectively. All the samples collected in this study were under the regulatory levels recommended.

## 3. Conclusions

The data collected in the present study from an experiment performed during different growing seasons in an industrial mill, through the contemporaneous application of different degermination processes to the same maize lot, underline the important role of the adopted milling process in the re-distribution of the FB content in milling products and by-products. As far as the by-products are concerned, the animal feed flour showed an important increase in contamination, in part because it receives cleaned fractions that are highly contaminated, while the germ resulted in a similar FB content to the whole kernel after cleaning. As far as the endosperm fractions are concerned, the FBs in products that are derived from the horny endosperm are distributed in a different way from those obtained from the floury endosperm. The former are less contaminated, and thus present a lower health risk in the food chain. Finally, this study has proved, for the first time, that the application of a TD process to dry milling leads to endosperm fractions with a lower health risk for fumonisin contamination than the DD process.

## 4. Material and Methods

### 4.1. Maize Milling Processes

The fate of FBs has been investigated by sampling and analysing commercial maize lots (>200 t), cultivated in 2011–2013 period in the same growing area in Northwest Italy. In each year, the sampling was replicated on 3 different lots, for a total number of sampled lots equal to 9 (Table 1). In order to obtain a high homogeneity during the milling process, the selected lots were constituted by a limited number of cultivars: 7 of the compared 9 lots were constituted by a single hybrid, which was chosen from among the ones commonly processed in the industrial mill, while the other 2 lots were mixtures of 2 or 3 of these representative hybrids

All the maize lots were processed in an industrial mill on two separate dry-milling lines based on different degermination processes as described in detail in Blandino et al. [8]. In both processes, maize kernels were cleaned using a dry stoner, an intensive horizontal scourer, a vibrating aspirator and an optical sorting equipment.

**Figure 1:**
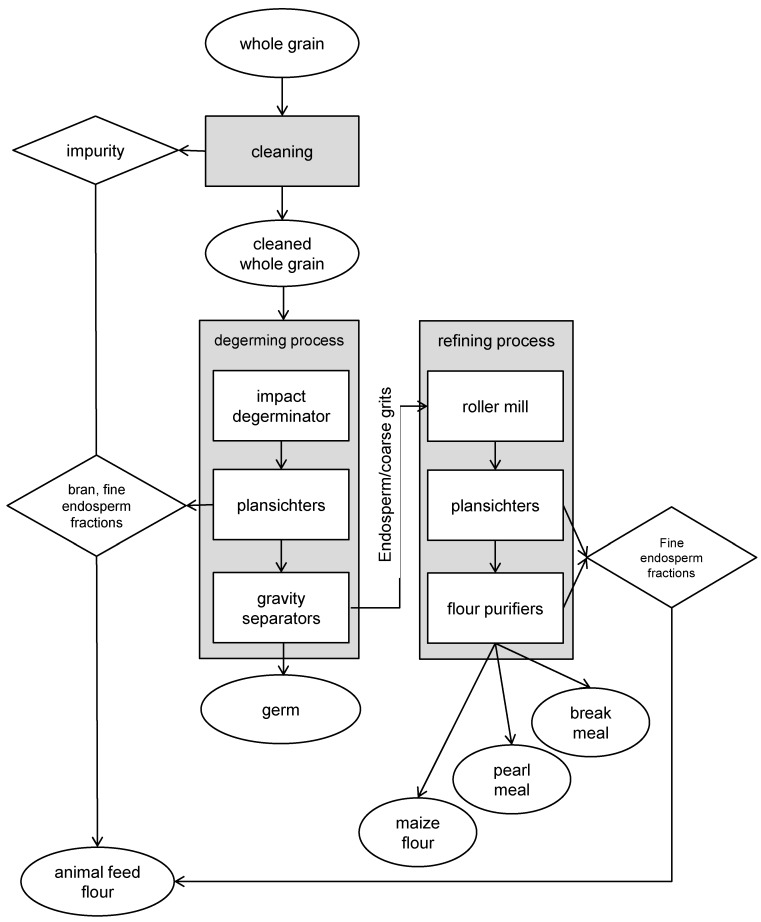
Flow diagram of dry milling with a dry degermination (DD) system. The raw materials, products and by-products collected and analysed in the study are reported in the oval.

The first process was based on a dry milling technology coupled to a dry-degermination (DD) system (Figure 1) and produced maize flour, pearl meal and break meal with different particle size, as showed in Table 2. Maize flour had 95% of particles size under 315 µm, while pearl meal and break meal differ because the first one had 82% of particles between 500–1000 µm, and break meal 70% and 27% between 315–500 µm and between 500–1000 µm, respectively. Taking into consideration the FB limit established by European Commission Regulation (EC) No. 1126/2007 [13], the maize flour was the only milling fraction with a particle size completely <500 µm and was therefore the only one with maximum level for the sum of FB_1_ and FB_2_ of 2000 µg kg^−1^. All the other milling fractions had a mean particle size >500 µm and a maximum limit for these toxins of 1400 µg kg^−1^.

**Figure 2:**
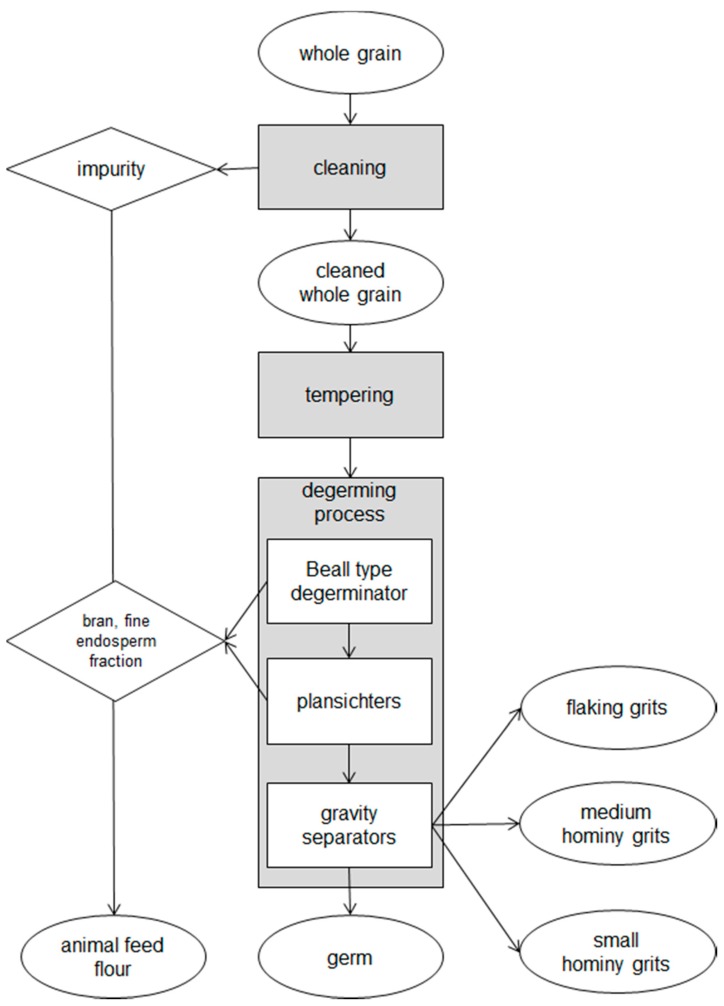
Flow diagram of dry milling with a tempering-degerming (TD) system. The raw materials, products and by-products collected and analysed in the study are reported in the oval.

The second process was based on a dry-milling technology coupled to a tempering-degermination (TD) system (Figure 2) and the endosperm was broken into large flaking grits, medium and small hominy grits (Table 5). Flaking grits were pieces of horny endosperm bigger than 4 mm, the medium and small hominy grits were constituted by pieces of endosperm of 3–4 mm and 2.5–3 mm for 73% and 60%, respectively. In the TD system after the cleaning of maize kernels the water was added to increase the moisture content to approximately 20% by adding 50–70 kg of water for each maize tonne, according to the moisture content of the stored kernels.

In both processes, the main by-products were the germ and the animal feed flour, a mixture of impurities, bran and a part of the mealy endosperm. The usual expected yield of these by-products, in comparison to whole grain before cleaning, was 10% for the germ and 35% for the animal feed flour.

### 4.2. Sampling

Samples of each dry milling product and by-product were drawn from opening slits of the plant and the adopted sampling procedure was derived from European Commission Regulation (EC) No 401/2006 [31]. Considering that each lot was of about 200 t and that the plant mills 5 t h^−1^ of maize kernel, a dynamic sampling procedure was planned in which each aggregate sample was the result of careful blending of 40 incremental samples of 100 g each, collected for 1 h at regular intervals. A sampling lasting 1 h was performed twice for each lot and each dry-milling process in order to obtain two replications. The samples collected for each lot were the whole grain before and after cleaning, all the products (break meal, pearl meal and maize flour for DD, flaking grits, medium and small hominy grits for TD) and the by-products (germ, animal meal) for both processes (DD and TD), for a total of 216 samples (3 years × 3 lots/year × 12 milling fractions × 2 replications). The sampled products (in the oval shapes in Figure 1 and Figure 2) were collected during milling so that the sum of the products of each process represented the lot of origin. The samples were maintained at −18 °C until the mycotoxin analysis was performed.

### 4.3. FB Analysis

#### 4.3.1. Sample Preparation, Extraction and Clean-Up

All the samples were subjected to a further milling step using a hammer mill (ZM 200 Ultra Centrifugal Mill, Retsch GmbH, Haan, Germany) to provide a homogenous particle dimension of less than 1 mm.

For the fumonisin extraction, 50 g of flour was mixed with 100 mL methanol/water (80:20, *v*/*v*) on a mechanical shaker (shaker mod. M102-OS, MPM Instruments, Milan, Italy) at 100 rpm for 20 min. The extracts were filtered through Whatman no. 1 filters (GE Healthcare Europe GmbH, Freiburg, Germany) and 10 mL of filtered extract were diluted with 40 mL of Phosphate Buffered Saline (PBS) pH 7.8 (Sigma-Aldrich, St. Louis, MO, USA). A second filtration was performed with a Munktell Glass Microfiber AB (Munktell Filter AB, Falun, Sweden).

The clean-up method was performed with a FumoniTest WB^®^ immunoaffinity column (VICAM^®^, Watertown, MA, USA). The clean-up procedure involved pipetting 10 mL of filtered extract and passing it completely through a FumoniTest WB^®^ affinity column at a rate of about 1–2 drops/second. After, 5 mL of PBS were added and passed through the column and at last the analyte was recovered with 2 mL of pure LC/MS (Liquid Chromatography/Mass Spectrometry) grade methanol and injected into the LC-MS/MS system, according to the method described below.

#### 4.3.2. LC-MS/MS Analysis

FB_1_ and FB_2_ were quantified by injecting 10 μL of the purified extracts into the LC-MS/MS system. The LC system consist of a Varian 212-LC chromatographic pump, a reversed-phase Agilent column, Pursuit 5 C18 (50 × 2.1 mm, 5 μm) and a ProStar 410 autosampler. The LC system was coupled with a triple quadrupole mass spectrometer 310-MS equipped with an electrospray ionization (ESI) source. The chromatographic run had a duration of 15 min (t_R_ FB_1_ = 4.9 min; t_R_ FB_2_ = 5.6 min), with acetonitrile and water acidified with acetic acid 0.1% as the mobile phase. The FBs were identified by using the electrospray ionization source in the positive ion mode. The protonated FB_1_ (722 *m*/*z*) molecule was fragmented into its product ions at 352 *m*/*z* (used for identification) and 334 *m*/*z* (used for quantification). For FB_2_ the fragmentation pathway was instead the production of the ions at 318 *m*/*z* (used for identification) and 336 *m*/*z* (used for quantification) from the precursor protonated FB_2_ (706 *m*/*z*). The quantification was performed on the basis of calibration curves with a linearity range of between 4 and 4000 µg kg^−1^. For both FB_1_ and FB_2_ the limit of detection (LOD) and the limit of quantification (LOQ) were 1 and 4 µg kg^−1^, respectively. The mean percentage of recovery at two different concentration levels (FB_1_ = 1800 and 3600 µg kg^−1^; FB_2_ = 600 and 1100 µg kg^−1^) for FB_1_ and FB_2_ was 78% (relative standard deviation, RSD%: 11%) and 87% (RSD%: 15%), respectively. All the reported results were corrected for the recovery rate.

### 4.4. Statistical Analysis

The normal distribution and homogeneity of variances were verified by performing a Kolmogorov-Smirnov normality test and a Levene test, respectively.

The FB contamination was compared by means of an analysis of variance (ANOVA), in which the milling fractions were the independent variables and the year of production of the processed maize lots was the random factor. Since the level of contamination within the maize produced in the same growing season was very similar, the lots cultivated and sampled in the same year were considered as replication. The FB content were transformed using the equation:

*y* = ln(*x* + 1) (1)

to normalize the residuals. Multiple comparison tests were performed, according to the Ryan-Einot-Gabriel-Welsh F (REGWF) post hoc test on treatment means. Statistical data analysis was carried out with the SPSS software package, version 24.0.

## Figures and Tables

**Table 1:** Maize lots processed in the industrial mill, ranked according to the maize production year and mean ± standard deviation (SD) of B-series fumonisins: FB_1_, FB_2_ and FBs in pre-cleaned whole grain.

Lots	Year	Hybrid	FB_1_ ± SD (µg·kg^−1^)	FB_2_ ± SD (µg·kg^−1^)	FBs ± SD (µg kg^−1^)
1	2011	DKC6795	407 ± 62	122 ± 5	529 ± 58
2	2011	Pioneer P1543	211 ± 192	91 ± 63	302 ± 255
3	2011	Pioneer 3245	170 ± 151	69 ± 70	239 ± 220
4	2012	Pioneer P1547	866 ± 331	354 ± 154	1220 ± 483
5	2012	Mixture of Pioneer P1543 and P1547	901 ± 285	385 ± 120	1286 ± 405
6	2012	Pioneer 3245	999 ± 721	369 ± 303	1368 ± 995
7	2013	Pioneer P1547	1418 ± 590	525 ± 203	1943 ± 685
8	2013	Pioneer P1758	1259 ± 76	420 ± 25	1678 ± 97
9	2013	Mixture of Pioneer 3245, P1543 and DKC6795	1979 ± 448	770 ± 266	2749 ± 706

The average FB_1_ and FB_2_ contaminations plus the sum of FB_1_ + B_2_ (FBs) of the pre- and post-cleaning of the whole grain and of all the fractions derived from the two milling processes are reported in Table 2. The reported values per year in Table 2 are based on 72 replications and refers to the average FB content of 12 milling fractions × 3 lots × 2 sampling repetitions.

**Table 2:** Fumonisin B_1_ (FB_1_), B_2_ (FB_2_) and sum of B_1_ + B_2_ (FBs) in milling fractions obtained from different dry milling processes on maize lots with different contamination levels.

Factor	Dry Milling Process ^a^	Source of Variation	FB_1_			FB_2_			FBs		
			T		N (µg kg^−1^)	T		N (µg kg^−1^)	T		N (µg kg^−1^)
Year	-	2011	4.6		255	3.7		84	4.9		340
	-	2012	6.1		718	5.1		295	6.4		1013
	-	2013	6.3		860	5.3		395	6.6		1255
Milling fraction	-	pre-cleaned whole grain	6.5	b	912	5.5	b	345	6.8	b	1257
	-	post-cleaned whole grain	5.8	c	534	4.8	c	191	6.1	c	725
	DD	germ	5.7	c	396	4.6	c	132	6.0	c	528
		animal feed flour	7.3	a	1718	6.5	a	872	7.7	a	2591
		maize flour	5.8	c	454	4.8	c	169	6.2	c	623
		break meal	4.8	de	159	3.9	de	57	5.1	de	216
		pearl meal	4.4	e	114	3.5	de	43	4.8	e	157
	TD	germ	5.7	c	386	4.6	c	124	6.0	c	510
		animal feed flour	7.5	a	2069	6.7	a	1018	7.9	a	3087
		small hominy grits	5.1	d	205	4.0	d	66	5.3	d	272
		medium hominy grits	4.5	e	109	3.5	e	38	4.8	e	147
		flaking grits	3.7	f	54	2.9	f	23	4.0	f	76
		*p*-value	<0.001		-	<0.001		-	<0.001		-
		SEM ^b^	1		-	0.9		-	0.9		-
Milling fraction × year		*p*-value	0.102		-	0.165		-	0.204		-

Means followed by different letters in a column are significantly different (the level of significance, *p*-value is shown in the table). The reported contamination means are means of transformed [T; *y* = ln(*x* + 1)] and not transformed (N) values. The reported values for the milling fraction are based on 18 replications (3 years × 3 lots × 2 repetitions), while the values per year are based on 72 replications (12 products or milling fractions × 3 lots × 2 repetitions). ^a^ dry milling process: DD, dry degermination; TD tempering degermination. ^b^ SEM = standard error of the means.

**Table 3:** Yield and FBs mass balance of the maize products after the milling processes.

Dry Milling Process ^a^	Products	Yield ^b^ (%)	FBs Mass Balance ^c^ (%)
DD	Germ	10	3.9
	Animal feed flour	35	67.3
	Maize flour	5	2.3
	Break meal	20	3.2
	Pearl meal	30	3.5
TD	Germ	10	4.4
	Animal feed flour	35	92.5
	Small hominy grits	7	2.1
	Medium hominy grits	19	2.4
	Flaking grits	29	1.6

^a^ Dry milling process: DD = dry degermination; TD = tempering degermination. ^b^ Yield of each product expressed as a percentage of the processed grain weight. ^c^ Mass balance calculated as the quantity expressed in percentage of FBs present in each milling product.

**Table 4:** Percentage of fumonisin B_1_ + B_2_ (FBs) remaining in each of the degermination milling fractions, with and without tempering, compared to the contamination of the whole kernel.

Degermination Process ^a^	Type of Mill	Milling Fractions ^b^	Particle Sizes (µm)	FBs ^c^ (%)	References
TD	Experimental	#12 grits	1680–2830	54	[16]
		#7 grits	2830–4000	42	
		#5 grits	>4000	15	
		special meal	<300	82	[15]
		semi coarse meal ^d^	710–2000	35	
		super meal	300–1400	15	
TD	Industrial	small hominy grits	1000–2000	22	Present manuscript
		medium hominy grits	2000–4000	12	
		flaking grits	>4000	6	
		small hominy grits	2500–3000	48	[17]
		medium hominy grits	3000–4000	10	
		flaking grits	> 4000	12	
		fine flour	<300	52	[18]
		coarse flour	300–850	12	
		flaking grits	>4000	8	
		flour		101	[21]
		Meal		29	
		flaking grits		14	
		flour		23	[27]
		Meal		10	
		Grits		9	
		flour		29	[16]
		flaking grits		8	
		flour		9	[14]
		small grits		17	
		large grits		8	
		special meal	<300	24	[15]
		super meal	300–1400	1	
		flour	21% > 180	15	[20]
		superior meal	31% > 500	12	
		extra meal	76% > 500	4	
		flour	<500	93	[28]
		grits	>500	6	
		endosperm		30	[29]
		corn meal		23	
		grits		11	
DD	Industrial	maize flour	<315	50	Present manuscript
		break meal	315–500 µm	17	
		pearl meal	500–1000 µm	13	
		maize flour	<350 µm	61	[17]
		break meal	350–500 µm	22	
		pearl meal	500–800 µm	15	
		maize flour	<500 µm	37	[11]
		pearl meal	500–1000 µm	11	
		maize flour		141	[30]
		cornmeal semolina		60	

^a^ Degermination process: DD = dry degermination; TD = tempering degermination. ^b^ According to the terminology and information on the particle size reported by the authors. ^c^ Percentages of contamination are compared to the whole kernel before the milling process. ^d^ Coarse meal with some pericarp, tip cap and germ fragments.

**Table 5:** Distribution of the particle sizes of the maize products after the milling process.

Dry Milling Process ^a^	Products	Particle Size Distribution (%)					
		<315 µm	315–500 µm	500–710 µm	710–800 µm	800–1000 µm	>1000 µm
DD	Maize flour	95	5				
	Break meal	3	70	20	5	2	
	Pearl meal	4	12	35	22	25	2
		<2500 µm	2500–3000 µm	3000–4000 µm	>4000 µm		
TD	Small hominy grits	40	60				
	Medium hominy grits	5	20	73	2		
	Flaking grits				100		

^a^ Degermination process: DD = dry degermination; TD = tempering degermination.

## Abbreviations

ANOVA	analysis of variance
FB_1_	fumonisin B_1_
FB_2_	fumonisin B_2_
FBs, B-series fumonisins	sum of FB_1_ and FB_2_
DD	dry degermination
TD	tempering-degermination
EC	European Commission
ESI	Electrospray ionization
LC	Liquid chromatography
MS	Mass spectrometry
PBS	Phosphate buffered saline
REGWF	Ryan-Einot-Gabriel-Welsh F test
